# Supplementary material for: Spatial biology of Ising-like synthetic genetic networks
Source: BMC Biol. 2023 Sep 4;21:185. doi: 10.1186/s12915-023-01681-4 (PMC10478219; doi:10.1186/s12915-023-01681-4)
Supplement: Supplementary file 3 — Additional file 3: Supplementary figures S13. Finite-size scaling analysis of Contact Process Ising Model (CPIM). Supplementary figures S14. Magnetization and Neighborhood size. Supplementary figures S15. Time series of colonies of spherical E. coli cells carrying the ferromagnetic system with reporter vector 1. Supplementary figures S16. Dynamics of reporter gene expression (spin flipping rates) in ferromagnetic colonies of spherical E. coli cells. Supplementary figures S17. Neighborhoods and Probability. Supplementary figures S18. Colony size expansion in a 3 point time series. Supplementary figures S19. Average size of spherical E. coli cells. Supplementary figures S20. Colonies of rod-shaped E. coli cells carrying the ferromagnetic system with reporter vector 1 or 2. Supplementary figures S21. Colonies of rod-shaped E. coli cells carrying the anti-ferromagnetic system with reporter vector 1 or 2. Supplementary figures S22. Colonies of spherical E. coli cells carrying the ferromagnetic system with reporter vector 1 or 2. Supplementary figures S23. Colonies of spherical E. coli cells carrying the anti-ferromagnetic system with reporter vector 1 or 2. [file 12915_2023_1681_MOESM3_ESM.pdf]

Spatial biology of Ising-like synthetic genetic networks

Kevin Simpson[1], Alfredo L'Homme[2], Juan Keymer[3,4,5*], Fernán Federici[1,2,6*]

[1]ANID – Millennium Science Initiative Program, Millennium Institute for Integrative Biology (iBio), Santiago, Chile.
[2]Institute for Biological and Medical Engineering, Schools of Engineering, Medicine and Biological Sciences, Pontificia Universidad Católica de Chile, Santiago, Chile.
[3]Institute for Advanced Studies, Shenzhen X-Institute, Shenzhen, China.
[4]Schools of Physics and Biology, Pontificia Universidad Católica de Chile, Santiago, Chile.
[5]Department of Natural Sciences and Technology, Universidad de Aysén, Coyhaique, Chile.
[6]FONDAP Center for Genome Regulation - Department of Molecular Genetics and Microbiology, Pontificia Universidad Católica de Chile, Santiago, Chile.

*Corresponding author(s). E-mail(s): juan.keymer@x-institute.edu.cn; ffederici@bio.puc.cl;
Contributing authors: kevinsimpson@gug.uchile.cl; ajlhomme@uc.cl ;

Keywords: Ising model, bi-stable, synthetic gene networks, spatial correlation, criticality

Additional file 3

Supplementary figures S13-S23 and tables S1-S7

List of Figures

S13 Finite-size scaling analysis of Contact Process Ising Model (CPIM) . . . 3
S14 Magnetization and Neighborhood size 4
S15 Time series of colonies of spherical *E. coli* cells carrying the ferromagnetic system with reporter vector 1 5
S16 Dynamics of reporter gene expression (spin flipping rates) in ferromagnetic colonies of spherical *E. coli* cells. 6
S17 Neighborhoods and Probability 7
S18 Colony size expansion in a 3 point time series 8
S19 Average size of spherical *E. coli* cells 9
S20 Colonies of rod-shaped *E. coli* cells carrying the ferromagnetic system with reporter vector 1 or 2 10
S21 Colonies of rod-shaped *E. coli* cells carrying the anti-ferromagnetic system with reporter vector 1 or 2 11
S22 Colonies of spherical *E. coli* cells carrying the ferromagnetic system with reporter vector 1 or 2 12
S23 Colonies of spherical *E. coli* cells carrying the anti-ferromagnetic system with reporter vector 1 or 2 13

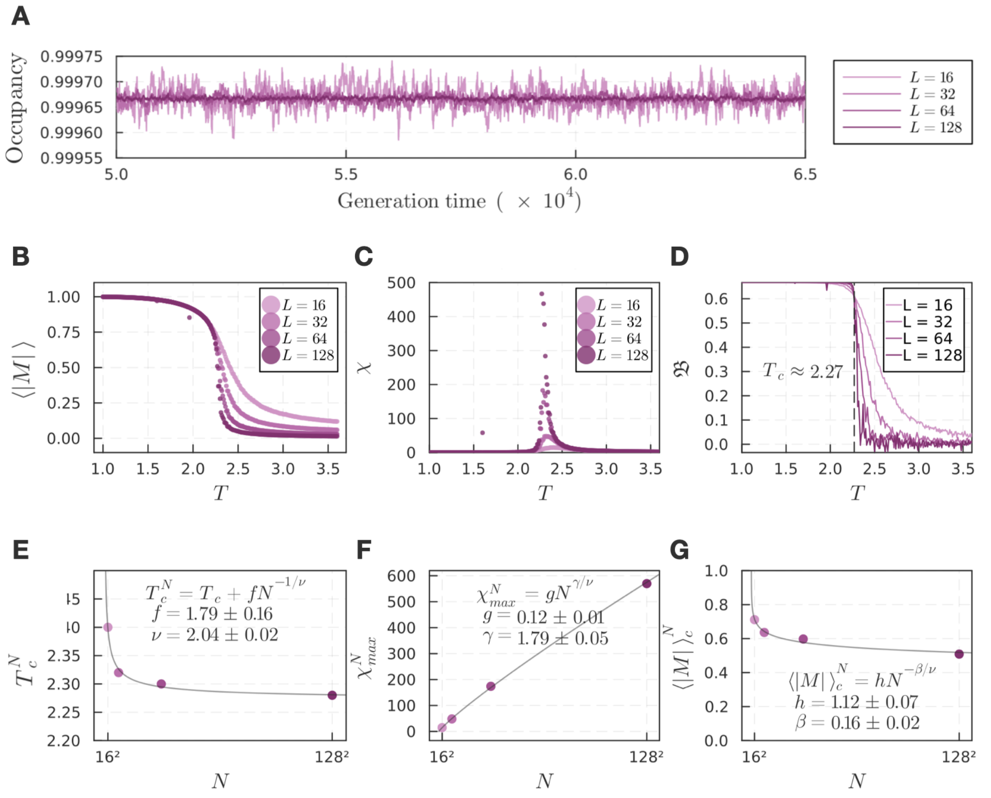

Fig. S13: **Finite-size scaling analysis of Contact Process Ising Model (CPIM)** (A) Lattice occupancy $1 - \varnothing/L^2$ (i.e. the density of occupied sites in the lattice) fluctuations over the generation times considered in this study, (B) average magnetization per site $\langle|M|\rangle$, (C) magnetic susceptibility per site χ, and (D) Binder cumulant $\mathfrak{B}$ vs temperature T for different lattice sizes $L = \{16, 32, 64, 128\}$. (E) Position of the magnetic susceptibility per site peak, T_c^N, (F) height of the magnetic susceptibility per site peak, X_{max}^N, and (G) average magnetization per site at the critical point $\langle|M|\rangle_c^N$ as a function of the system size $N = L^2$. All data points are averages over 15,000 generations and 10 simulations (replicates) using the model parameters $b = 0.03$, $d = 0.00001$, and $\alpha = 0.1$.

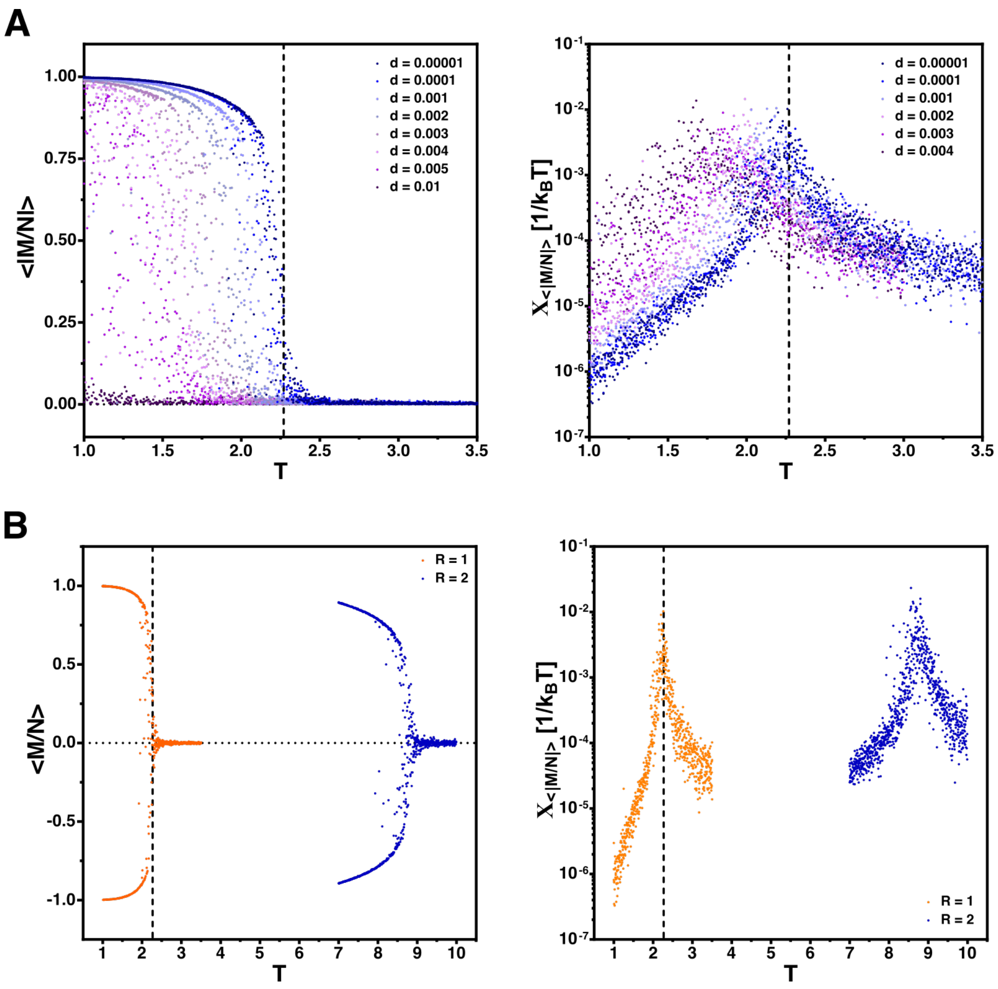

Fig. S14: **Behavior of the critical value of T for different parameters of CPIM.** A) Absolute values of the time-averaged magnetization per site $<|M/N|>$ (left) and magnetic susceptibility per site $X_{<|M/N|>}$ (right) as a function of T for ferromagnetic populations simulated with the CPIM with nearest neighbors at different values of death rate d. Birth rate = 0.03, differentiation rate = 0.1, lattice = 256x256. Dotted vertical lines mark the critical value of T of the Ising model ($T_c = 2.27$). (B) Time-averaged magnetization per site $< M/N >$ (left) and magnetic susceptibility per site $X_{<|M/N|>}$ (right) as a function of T for ferromagnetic populations simulated with CPIM for two neighborhoods (R = 1: nearest neighbors, R = 2: next nearest neighbors). For these simulations the following parameters were used: birth rate = 0.03, death rate = 0.00001, differentiation rate = 0.1, lattice = 256x256. 3 simulations per each value of T were used, and to obtain the time average of the magnetization per site, 11 values were taken between generations 6800 and 9800.

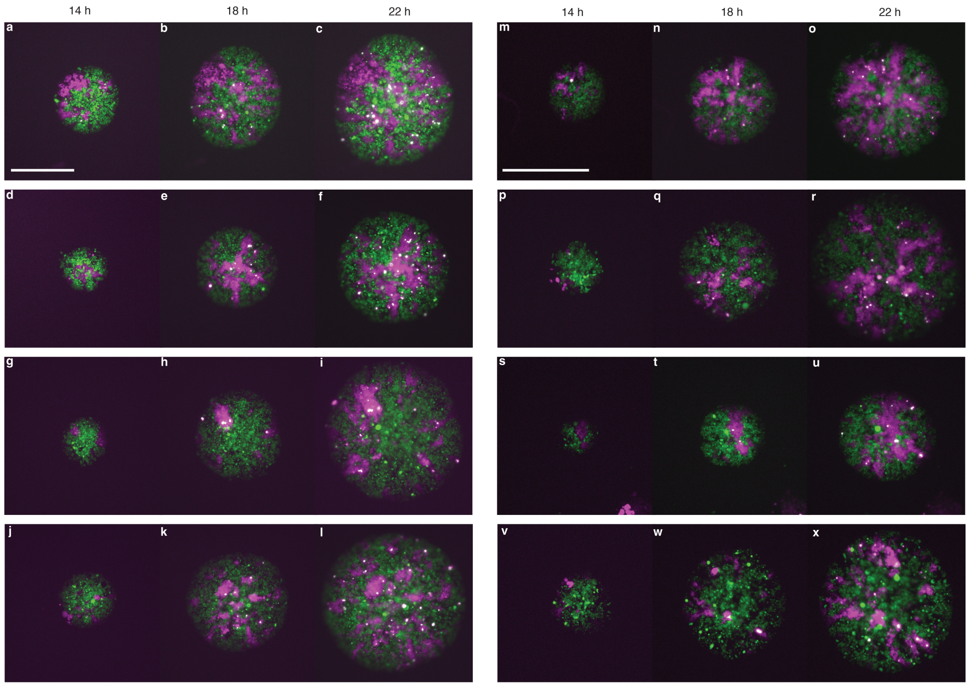

Fig. S15: **Time series of colonies of spherical *E. coli* cells carrying the ferromagnetic system with reporter vector 1.** Cells were grown on solid M9-glucose medium supplemented with 10^{-8} M of C6HSL, and images were taken 14, 18, and 22 hours after inoculation on the medium. Scale bars 100 μm.

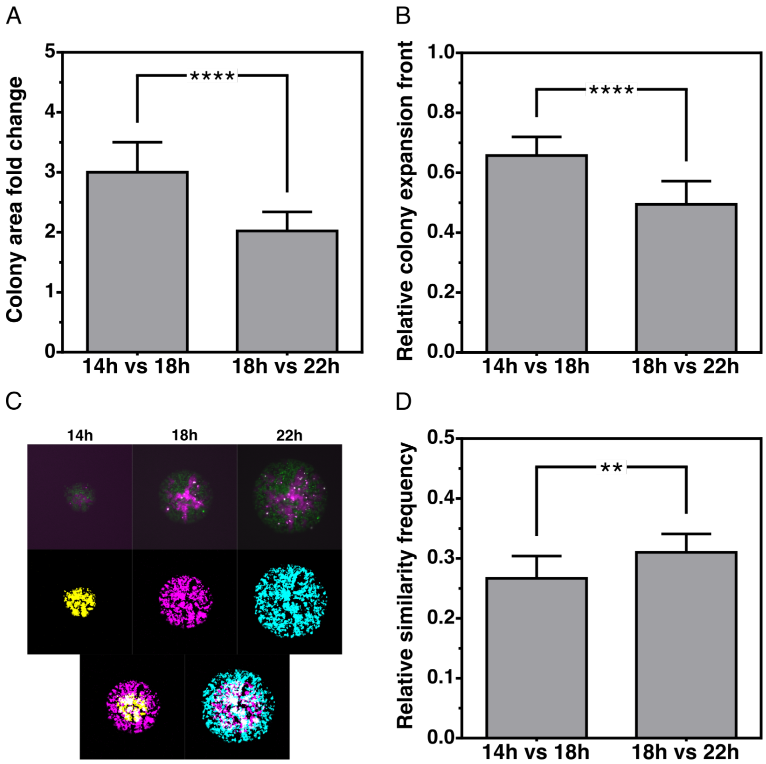

Fig. S16: **Dynamics of reporter gene expression (spin flipping rates) in ferromagnetic colonies of spherical *E. coli* cells.** A) Increase of the size of the colonies between 14 and 18 hours, and between 18 and 22 hours. To calculate the colony area fold change the area of the colonies at 18 (22) hours was divided by the area of the colonies at 14 (18) hours. B) Increase in the size of the expansion front of the colonies between 14 and 18 hours, and 18 and 22 hours. The relative colony expansion front corresponds to the area of the colonies at 18 (22) hours minus the area of the colonies at 14 (18) hours, and divided by the area of the colonies at 18 (22) hours. C) Example of the analysis of the temporal evolution of the patterns that emerge in the colonies. Images were taken at 14, 18, and 22 hours post-inoculation in M9-glucose medium supplemented with 10^{-8} M of C6HSL. Top: Merge of the red and green channels, Mid: Binarization of the green channel (for comparison, 14 hours is represented in yellow, 18 hours in magenta, and 22 hours in cyan), Bottom: Merge of the green channels at 18h and 22h, and 14h and 18h. D) Change in reporter expression. Quantification of the similarity between patterns observed at 14 and 18 hours, and 18 and 22 hours. The relative similarity frequency corresponds to the white pixels in C) (where cyan = magenta or magenta = cyan) divided by the total non-black pixels: white/(cyan + magenta + white) or white/(yellow + magenta + white). 13 colonies were used for the analysis, and statistical analysis was performed using unpaired two-tailed Mann-Whitney test ($\alpha = 5\%$). $**$: P value = 0.0019, $****$: P $\leq$ 0.0001.

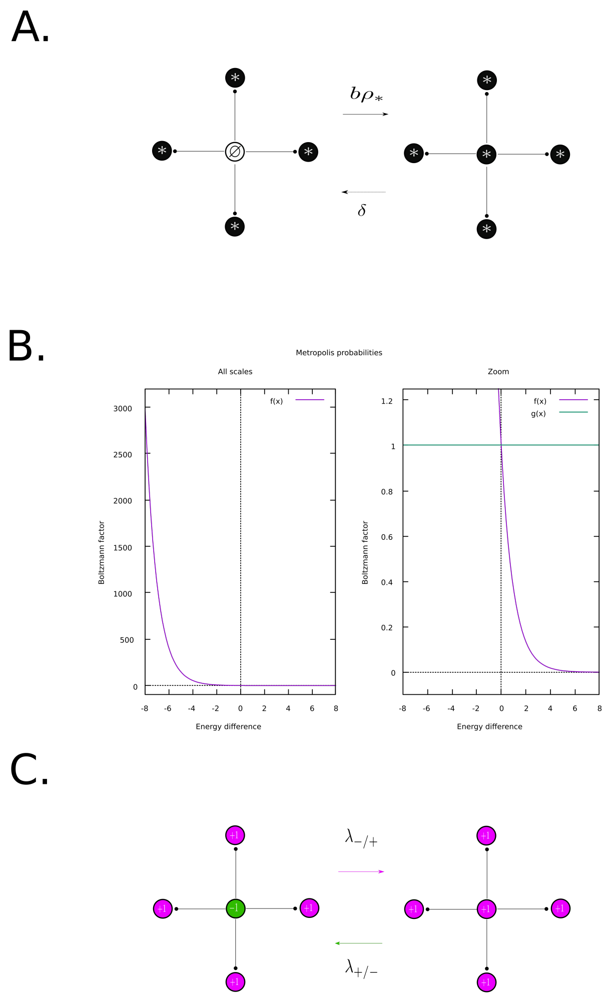

Fig. S17: **Neighborhoods and Probability.** A) Nearest Neighborhood configuration with a vacant (focal) site with fully occupied surroundings, which can be colonized at rate b as $\rho_* = 1$, or a reverse situation where a fully surrounded occupied site goes extinct at rate δ. B) Boltzmann factor as a function of energy difference for a proposed flip of a spin. C) Nearest Neighborhood configuration with a single spin down state fully surrounded by spin up states, and with forward spin flip probabilities being $\lambda_{-/+}$ and $\lambda_{+/-}$ for a backwards flip.

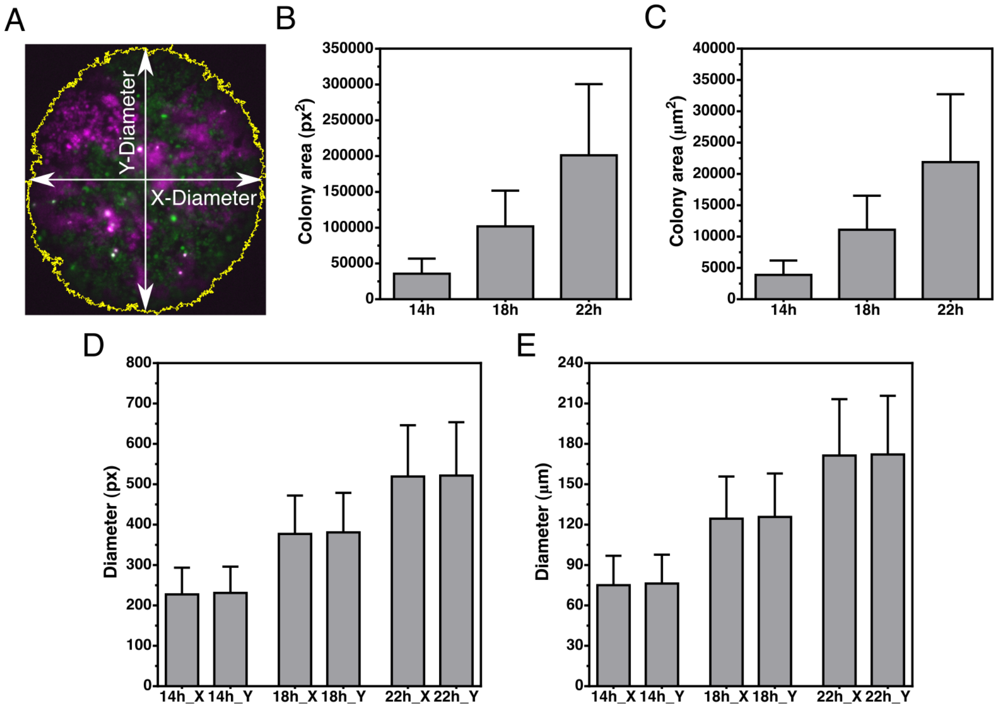

Fig. S18: **Colony size expansion in a 3-point time series.** A) Example of a colony and the quantification of its area, x-Diameter, and y-Diameter. B-C) Average size of the colonies in square pixels (B) or square micrometers (C) at 14h, 18h, and 22h post-inoculation. D-E) Average x-Diameter and y-Diameter of the colonies in pixels (D) or micrometers (E) at 14, 18, and 22 hours after inoculation on solid M9-glucose medium. Values and error bars correspond to the mean ± the standard deviation of data obtained from 13 colonies.

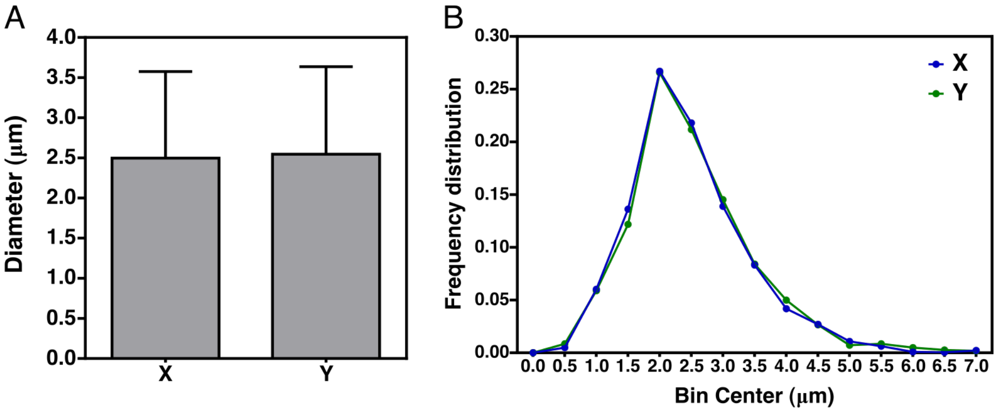

Fig. S19: **Average size of spherical *E. coli* cells.** A ferromagnetic colony of the *E. coli* KJB24 strain was re-suspended in M9-glucose medium. This strain contains a mutation in the cell wall protein RodA, generating spherical cells. Cells were observed under the microscope and the average (A) and frequency distribution (B) of the x- and y-diameter of the cells were calculated using the Analyze particles command of the Fiji distribution of ImageJ. Values and error bars in A correspond to the mean ± the standard deviation of data obtained from 2225 cells.

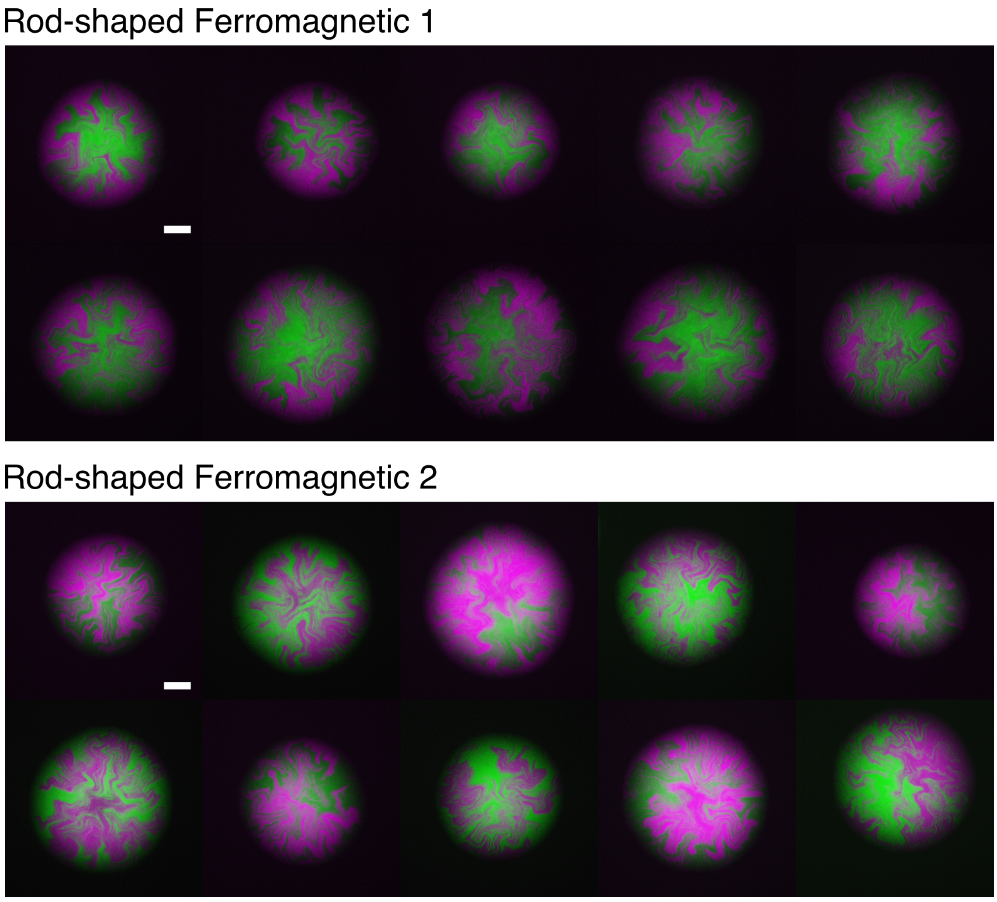

Fig. S20: **Colonies of rod-shaped *E. coli* cells carrying the ferromagnetic system with reporter vector 1 or 2.** Cells were grown on solid M9-glucose medium supplemented with 10^{-8} M of C6HSL. Images were taken approximately 14 hours after inoculation. Scale bars 100 μm.

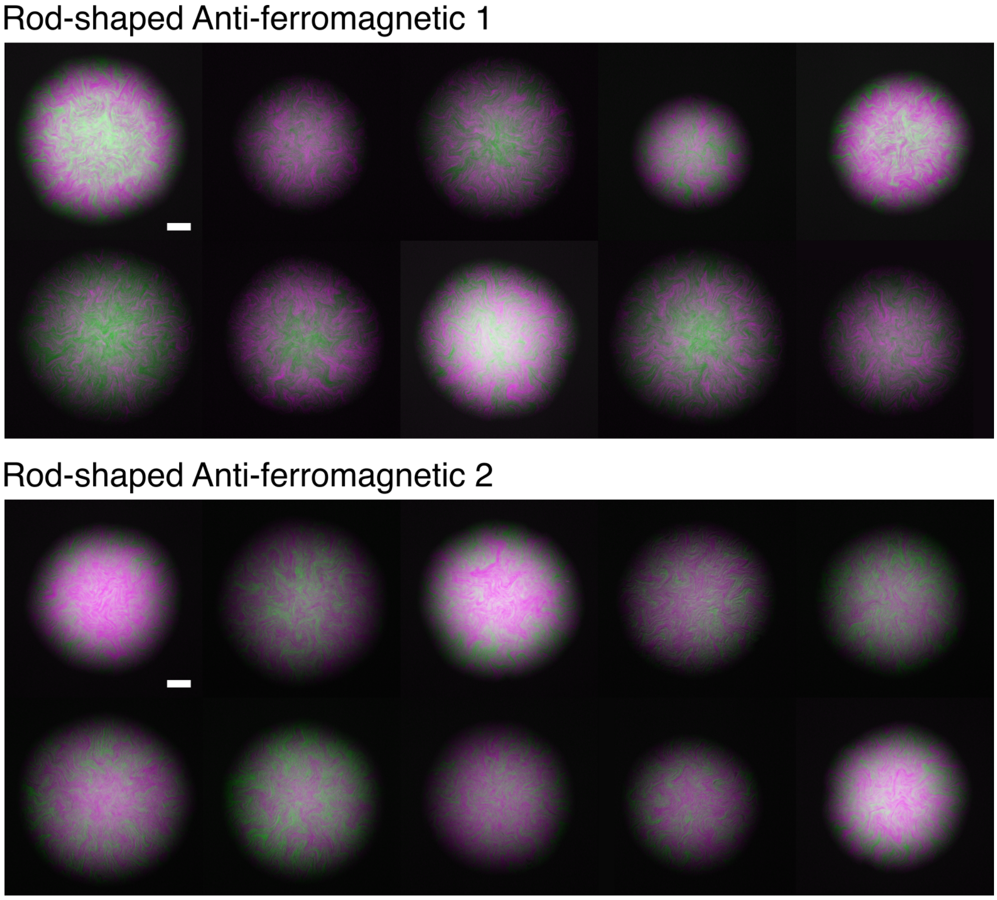

Fig. S21: **Colonies of rod-shaped *E. coli* cells carrying the anti-ferromagnetic system with reporter vector 1 or 2.** Cells were grown on solid M9-glucose medium supplemented with 10^{-8} M of C6HSL. Images were taken approximately 14 hours after inoculation. Scale bars 100 μm.

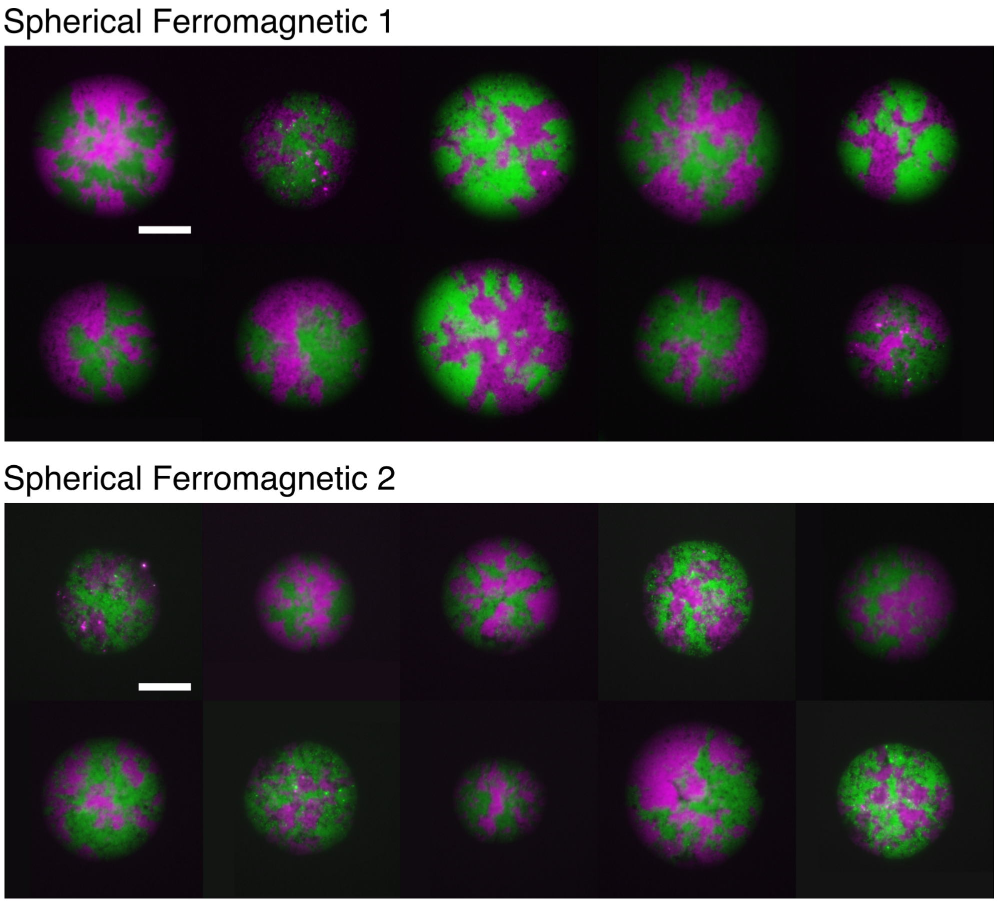

Fig. S22: **Colonies of spherical *E. coli* cells carrying the ferromagnetic system with reporter vector 1 or 2.** Cells were grown on solid M9-glucose medium supplemented with 10^{-8} M of C6HSL. Images were taken approximately 18 hours after inoculation. Scale bars 100 μm.

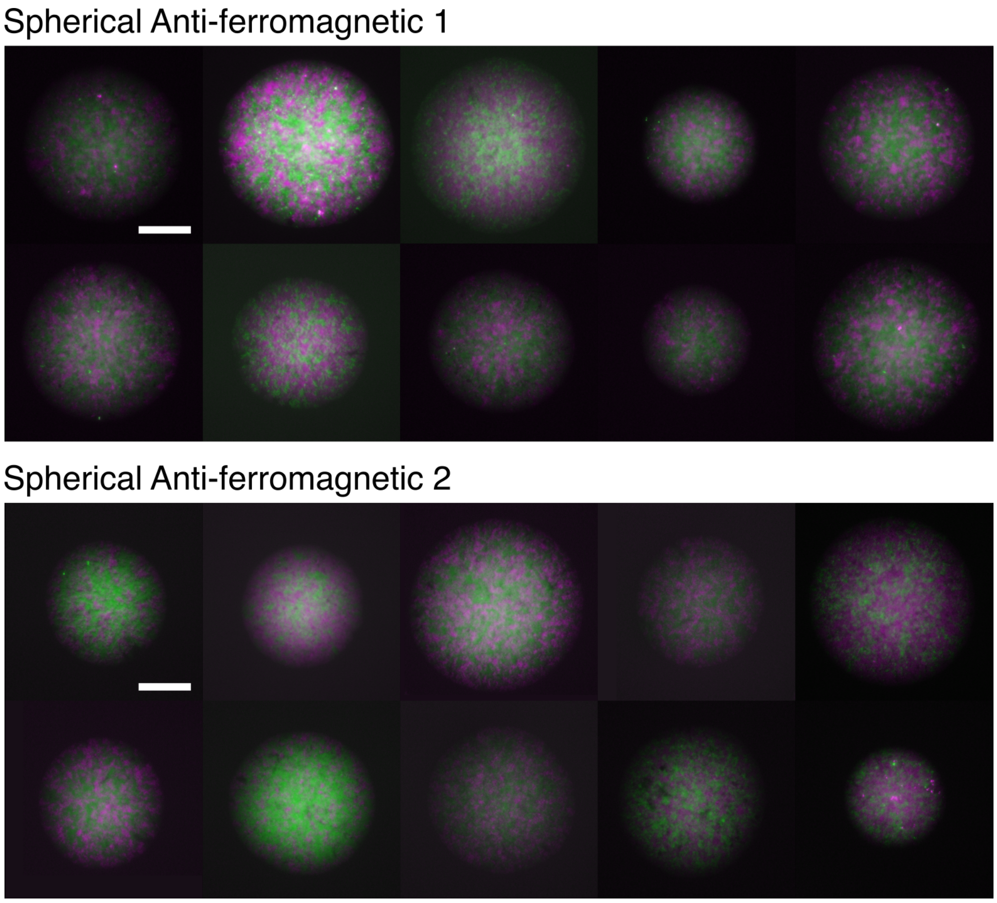

Fig. S23: **Colonies of spherical *E. coli* cells carrying the anti-ferromagnetic system with reporter vector 1 or 2.** Cells were grown on solid M9-glucose medium supplemented with 10^{-8} M of C6HSL. Images were taken approximately 18 hours after inoculation. Scale bars 100 μm.
